# HybridMolGen: a unified framework for goal-directed molecular generation via multi-objective reinforcement learning

**DOI:** 10.1093/bioinformatics/btag170

**Published:** 2026-04-04

**Authors:** Masoud Amiri, Zahra Nasirinia

**Affiliations:** Department of Biomedical Engineering, School of Medicine, Kermanshah University of Medical Sciences, Kermanshah, 6715847141, Iran; Department of Biomedical Engineering, School of Medicine, Kermanshah University of Medical Sciences, Kermanshah, 6715847141, Iran

## Abstract

**Motivation:**

*De novo* molecular design remains a fundamental challenge in drug discovery, requiring simultaneous optimization of multiple conflicting objectives such as drug-likeness, synthetic accessibility, and novelty while maintaining chemical validity. We present HybridMolGen, a novel unified framework that synergistically combines three complementary deep learning paradigms: (1) diffusion probabilistic models that generate high-quality, chemically valid molecular samples through gradual noise removal, (2) SE(3)-equivariant graph neural networks that enforce geometric and topological constraints ensuring structural validity and molecular diversity, and (3) property-conditioned transformers that enable fine-grained control over multiple objectives through multi-layer cross-attention modulation.

**Results:**

These components operate within a multi-objective reinforcement learning paradigm that discovers optimal property tradeoffs without manual weight tuning. Extensive benchmarking on MOSES, GuacaMol, and ZINC-250k datasets demonstrates state-of-the-art performance: 99.7% validity, 94.3% novelty, average QED score of 0.753, and 4.9% improvement in GuacaMol overall scores. Critically, HybridMolGen discovers 1.57× more molecules satisfying all target criteria simultaneously (91.3% versus 58.3% for CPRL) and generates 2.23× more Pareto-efficient solutions compared to traditional scalarization, demonstrating genuine architectural synergy beyond simple component aggregation. Comprehensive ablation studies confirm that the three-way integration outperforms even the best two-component combination by 6.5%, positioning HybridMolGen as a powerful tool for accelerating drug discovery pipelines.

**Availability and implementation:**

Implementation code is available as Supplementary Material, available as supplementary data at *Bioinformatics* online.

## 1 Introduction

The development of novel therapeutic compounds represents one of pharmaceutical science’s most formidable challenges. Traditional drug discovery pipelines require 10–15 years and investments exceeding $2 billion USD, with failure rates above 90% in clinical trials (Paul et al. 2010, DiMasi et al. 2016). This inefficiency stems fundamentally from the vastness of drug-like chemical space, estimated to contain between 10^23^ and 10^60^ synthesizable organic molecules (Bohacek et al. 1996, Polishchuk et al. 2013). Even with modern high-throughput screening capabilities testing millions of compounds annually, this represents an infinitesimal fraction of possible structures. Computational approaches offer a transformative alternative: rather than exhaustively searching existing compound libraries, generative artificial intelligence can design novel molecules optimized for specific therapeutic objectives (Olivecrona et al. 2017, Segler et al. 2018).

Despite significant progress in deep generative modeling for molecules (Gómez-Bombarelli et al. 2018, Jin et al. 2018, Brown et al. 2019, Polykovskiy et al. 2020), fundamental gaps persist in current methodologies. Existing approaches typically excel in isolated aspects—autoregressive models ensure validity but lack global coherence (Jin et al. 2018, Shi et al. 2020), diffusion models generate high-quality samples but provide limited property control (Hoogeboom et al. 2022, Xu, et al. 2023, Morehead and Cheng 2024), reinforcement learning methods optimize objectives effectively but may sacrifice molecular diversity (Olivecrona et al. 2017, You et al. 2018). More critically, most frameworks address molecular generation as single-objective optimization or employ naive scalarization for multiple properties (Fu et al. 2020), requiring extensive manual weight tuning and discovering only singular points on the Pareto frontier rather than the full spectrum of optimal tradeoffs (Zhang et al. 2024). The field lacks a unified architecture that simultaneously achieves: (1) high-quality sample generation matching training distributions, (2) precise control over multiple molecular properties, (3) principled multi-objective optimization without manual hyperparameter tuning, and (4) guaranteed chemical validity with structural diversity.

To address these multifaceted limitations, we introduce HybridMolGen, which achieves genuine architectural synergy through careful integration of complementary deep learning paradigms. Our framework combines three key strengths: diffusion probabilistic models (Ho et al. 2020) excel at generating high-quality, chemically valid samples with smooth exploration of molecular space; SE(3)-equivariant graph neural networks (Satorras et al. 2020 enforce geometric and topological constraints ensuring structural validity while maintaining molecular diversity; and property-conditioned transformers (Vaswani et al. 2017) enable fine-grained control over multiple objectives through multi-layer cross-attention modulation. This three-way architectural integration transcends the individual limitations of component methods, demonstrated by a 6.5% performance improvement over the best two-component combination, confirming genuine synergy rather than simple aggregation. Furthermore, our Pareto-based multi-objective reinforcement learning framework (While et al. 2006, Zhang et al. 2024) automatically discovers diverse optimal tradeoffs without manual weight tuning, generating 2.23× more Pareto-efficient solutions compared to traditional scalarization approaches. These innovations position HybridMolGen as a comprehensive solution for goal-directed molecular generation in drug discovery.

## 2 Related work

### 2.1 Molecular generation: from early approaches to modern diffusion models

Molecular generation has evolved through multiple complementary paradigms, each addressing specific aspects of the generation problem with distinct tradeoffs. Early approaches utilized variational autoencoders (VAEs) (Kingma et al. 2014, Gómez-Bombarelli et al. 2018) and generative adversarial networks (GANs) (Goodfellow et al. 2014, De Cao et al. 2018a, 2018b), which offered flexibility in learning molecular distributions but suffered from training instability and produced chemically invalid structures. To address validity concerns, graph-based methods such as Junction Tree VAE (Jin et al. 2018) and GraphAF (Shi et al. 2020) emerged, achieving superior chemical validity through structured generation that respects molecular constraints and chemical rules. Autoregressive models (De Cao et al. 2018b, Chang et al. 2018) provide sequential validity guarantees but struggle with long-range dependencies and global molecular coherence.

More recently, diffusion probabilistic models have become the state-of-the-art approach for high-quality molecular generation. Equivariant diffusion models (EDMs) (Hoogeboom et al. 2022) introduced diffusion to 3D molecular generation using SE(3)-equivariant graph neural networks, respecting the geometric structure of molecules. Building on this foundation, geometry-complete diffusion (GCDM) (Morehead and Cheng 2024) incorporated explicit geometric features, achieving state-of-the-art performance on molecular benchmarks. Geometric latent diffusion (GeoLDM) (Xu et al. 2023) further improved computational efficiency through latent space diffusion. Despite these advances in individual methodologies, existing single-modality approaches remain fundamentally limited in their ability to simultaneously optimize multiple conflicting objectives while maintaining structural diversity and exploration capability. HybridMolGen directly addresses each of these limitations through targeted architectural integration. First, unlike VAEs and GANs that suffer from training instability and chemically invalid structures, our diffusion-based generator with SE(3)-equivariant GNNs achieves 99.7% validity by enforcing geometric constraints during denoising. Second, while graph-based methods like JT-VAE ensure local validity, they lack global property control—our transformer-based cross-attention conditioning enables precise multi-property optimization across entire molecular structures. Third, unlike autoregressive models struggling with long-range dependencies, our diffusion process operates holistically on molecular graphs, maintaining global coherence. Fourth, where existing diffusion models (EDM, GCDM, GeoLDM) provide limited property control, our multi-layer transformer conditioning modulates GNN features at each of 12 layers via cross-attention, enabling fine-grained property steering. Finally, while prior multi-objective approaches rely on scalarization discovering only single Pareto points, our Pareto-based RL framework discovers 2.23× more frontier solutions automatically, eliminating manual weight tuning.

### 2.2 Multi-objective optimization: scalarization versus Pareto-based approaches

Multi-objective optimization is central to practical drug discovery, where conflicting goals must be balanced: maximizing drug-likeness and binding affinity while minimizing synthetic complexity and toxicity risks. Traditional approaches employ scalarization (Miettinen 1998, Fu et al. 2020), converting multiple objectives into a single weighted sum for optimization. While computationally efficient, scalarization suffers from fundamental limitations: (1) it discovers only singular points on the Pareto frontier per training run, requiring repeated retraining with different weight combinations to explore tradeoffs, and (2) manual weight specification is unintuitive and problem-specific, making the approach impractical for chemists.

Pareto-based approaches (Deb et al. 2002, While et al. 2006, Zhang et al. 2024) explicitly maintain diverse solutions along the frontier, capturing the full spectrum of optimal tradeoffs. However, these methods are computationally expensive and difficult to integrate with modern deep learning frameworks. Multi-objective reinforcement learning (MORL) (Roijers et al. 2013, Van Moffaert et al. 2014, Hayes et al. 2022) offers a promising middle ground, providing principled frameworks for discovering diverse policies without manual weight tuning. However, existing MORL applications often revert to scalarization in practice or achieve limited frontier coverage. Our work demonstrates that explicit Pareto-based ranking (While et al. 2006) combined with rank-based reward assignment within a reinforcement learning framework (Schulman et al. 2017) can efficiently generate molecules spanning the entire Pareto frontier, enabling medicinal chemists to select compounds matching project-specific constraints without model retraining.

### 2.3 Hybrid architectures and synergistic integration

Recent work increasingly recognizes that individual modeling paradigms possess inherent limitations that can benefit from complementary architectural integration. In molecular generation specifically, several hybrid approaches have emerged: MolGPT (LeCun et al. 2015) combined GPT-style transformers with molecular graphs for conditional generation, while Uni-Mol (Zhou et al. 2023) integrated transformers with 3D molecular representations achieving improved property prediction. MoleculeSTM (Liu et al. 2023) demonstrated successful integration of language models with molecular graphs through contrastive learning for multi-modal molecular understanding. For diffusion-based molecular design, DiffSBDD (Schneuing et al. 2023) combined diffusion models with protein pocket conditioning for structure-based drug design, and TargetDiff (Guan et al. 2023) integrated SE(3)-equivariant networks with target-aware generation for improved binding affinity. DrugGPT (Li et al. 2023) recently combined large language models with molecular generation for interactive drug discovery. However, these approaches either focus on single objectives or employ simple concatenation of components without achieving genuine architectural synergy through cross-attention mechanisms. Our contribution demonstrates how diffusion models (for generation quality), equivariant GNNs (for structural awareness), and transformers (for property control) can be integrated through multi-layer cross-attention, yielding a 6.5% performance improvement over the best two-component combination—empirical evidence of true architectural synergy.

## 3 Methodology

### 3.1 Problem formulation

We formulate goal-directed molecular generation as multi-objective optimization. Let *M* denote the set of valid molecules. The objective is:


$$\mathrm{maximize}\;f(m)=(f_{1}(m),f_{2}(m),\;\ldots ,\;f_{n}(m))\;\mathrm{subject}\;\mathrm{to}\;m\in M$$

where *f* represents molecular properties:

- *f*_1_: QED (drug-likeness) (Bickerton et al. 2012)
- *f*_2_: synthetic accessibility (SA) (Ertl and Schuffenhauer 2009) (inverted for optimization)
- *f*_3_: novelty (dissimilarity to training data)
- *f*_4_: property-specific constraints

Our approach balances two objectives: (1) generating novel molecules not in training data (exploration), and (2) maintaining valid drug-like distributions (exploitation). This is achieved by optimizing novelty while constraining LogP and MW to match training distributions.

### 3.2 HybridMolGen architecture overview

**Figure 1:**
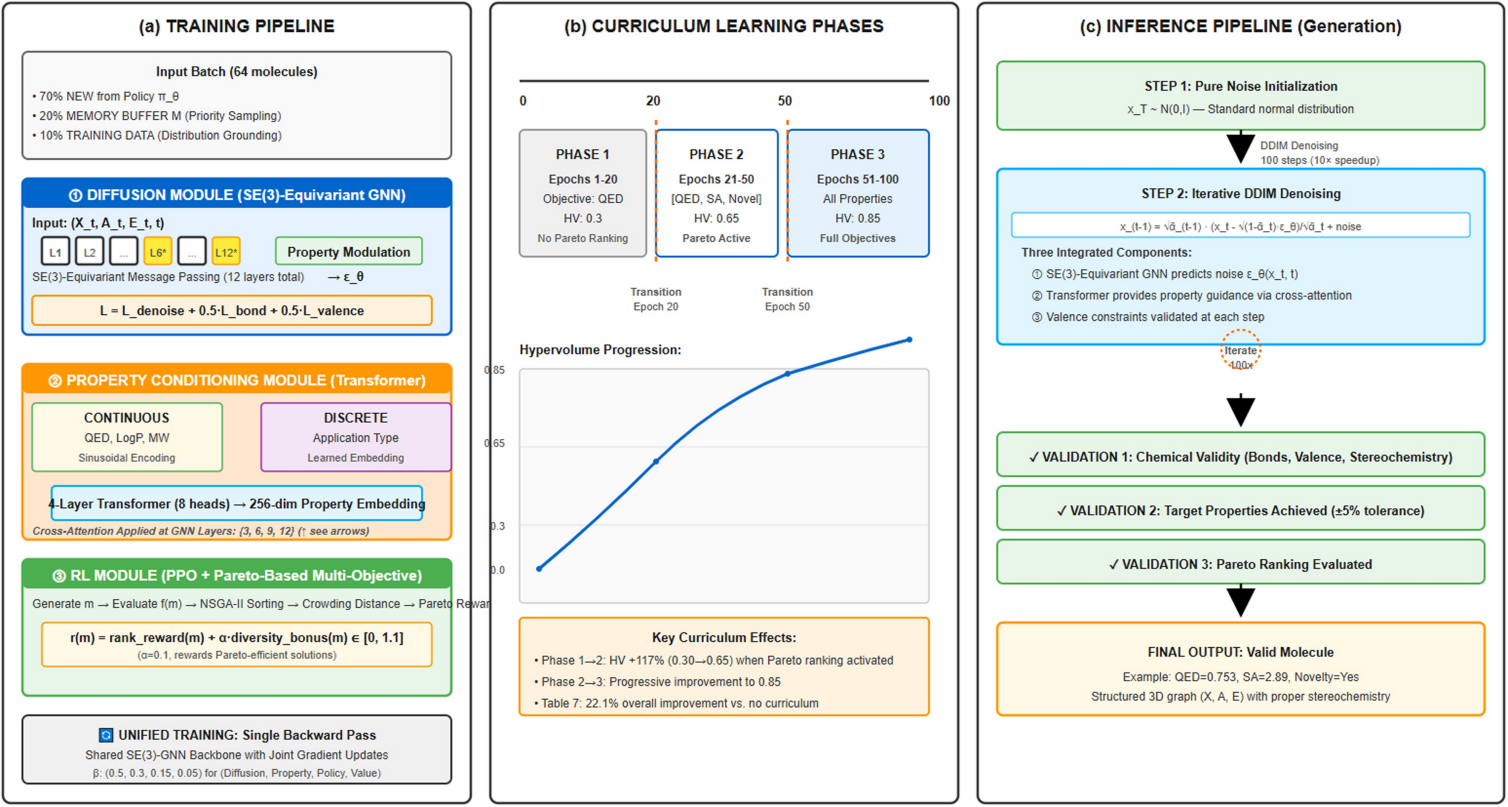
HybridMolGen architecture overview. (a) Training pipeline: input batch composition (70% new from policy, 20% memory buffer, 10% training data) flows through three integrated modules—the diffusion module with 12-layer SE(3)-equivariant GNN predicting noise *ε*_*θ*, the property conditioning module using 4-layer transformer with eight attention heads producing 256-dim embeddings applied via cross-attention at layers {3, 6, 9, 12}, and the RL module implementing PPO with Pareto-based rewards $r(m)\;=\;{\mathrm{rank}}_{\mathrm{reward}}(m)\;+\;\alpha \cdot {\mathrm{diversity}}_{\mathrm{bonus}}(m)$. All modules share a unified backward pass with joint gradient updates. (b) Curriculum learning phases: three-phase training strategy showing Phase 1 (epochs 1–20, QED only, HV = 0.3), Phase 2 (epochs 21–50, Pareto ranking activated, HV = 0.65), and Phase 3 (epochs 51–100, full objectives, HV = 0.85), achieving 22.1% overall improvement versus no curriculum. (c) Inference pipeline: generation from pure noise $x_{T}\;∼\;N(0,I)$ through iterative DDIM denoising (100 steps, 10× speedup) with three integrated components, followed by validation checks for chemical validity, target properties (±5% tolerance), and Pareto ranking evaluation.

HybridMolGen (Fig. 1) comprises three tightly integrated modules operating synergistically during both training and inference: (1) a diffusion-based generator (Ho et al. 2020) with SE(3)-equivariant graph neural networks (Satorras et al. 2021) for structural generation, (2) a property conditioning module using transformers (Vaswani et al. 2017) and cross-attention for multi-property control, and (3) a multi-objective reinforcement learning module (While et al. 2006, Schulman et al. 2017, Zhang et al. 2024) with Pareto-based optimization for tradeoff discovery. These components are trained jointly through iterative refinement with feedback loops, and connected during inference through a generation pipeline that ensures chemical validity and property satisfaction.

The architecture demonstrates genuine synergy through integrated training: the diffusion module generates candidate molecules, the property conditioning module guides generation toward target properties through cross-attention modulation at layers {3, 6, 9, 12}, and the RL module provides Pareto-based rewards combining rank-based scores with diversity bonuses (*α* = 0.1) to optimize multiple conflicting objectives simultaneously. The inference flow demonstrates the generation pipeline where noise is progressively denoised using DDIM (Song et al. 2021) with 100 steps (reducing from 1000) for 10× speedup, while maintaining property constraints through transformer conditioning (Vaswani et al. 2017), ultimately producing chemically valid molecules that satisfy multiple objectives. The SE(3)-equivariant architecture (Satorras et al. 2021) ensures rotational and translational invariance throughout the generation process.

#### 3.2.1 Diffusion-based generator with equivariant GNNs

The diffusion component implements denoising diffusion probabilistic models (Ho et al. 2020) operating jointly on molecular graphs. Molecules are represented as tuples (X, A, E) where $X\;\in \;R^{n\times 3}$ denotes 3D atomic Cartesian coordinates (x, y, z positions in Å), essential for capturing spatial relationships that determine molecular properties such as binding affinity and stereochemistry. $A\;\in \;{\left\{C,\;N,\;O,\;F,\;S,\;Cl\right\}}^{n}$ represents atom types (*n* atoms from six common organic elements). $E\;\in \;R^{n\times n\times 8}$ contains edge features comprising: (1) bond type one-hot encoding (single, double, triple, aromatic; four dimensions), (2) bond distance in Å (one dimension), (3) bond angle cosine relative to neighboring bonds (one dimension), and (4) conjugation and ring membership flags (two dimensions). The 3D representation enables SE(3)-equivariance, ensuring generated molecules respect rotational and translational invariance—critical for maintaining chemically meaningful geometric relationships during generation.

**Figure 2:**
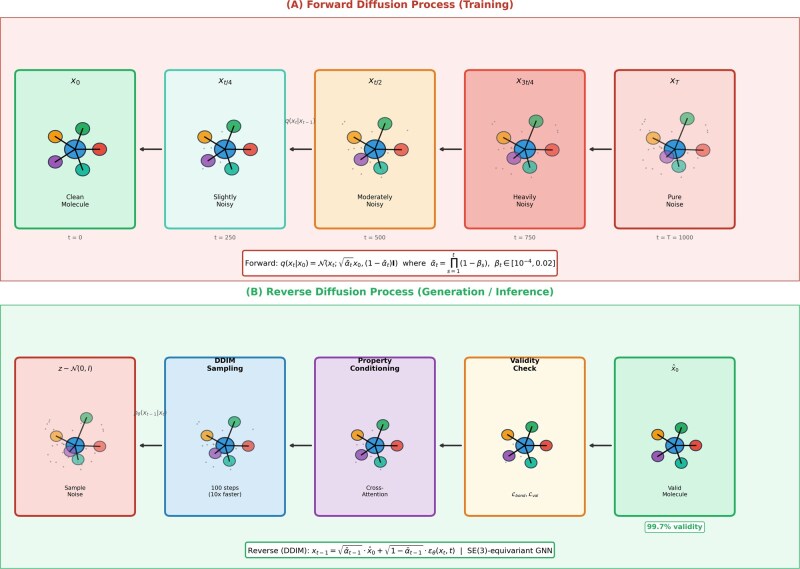
Forward and reverse diffusion process for molecular generation. (Top) Forward process gradually corrupts a clean molecule *x*_0_ over *T* = 1000 timesteps following a cosine variance schedule $\beta_{t}\;\in \;[10-4,\;0.02]$, transforming it into pure Gaussian noise *x_t_*. (Bottom) Reverse process learns to denoise, starting from sampled noise z ∼ N(0,I). The SE(3)-equivariant GNN predicts noise $\varepsilon_{\theta }(x_{t},\;t)$ at each timestep. DDIM sampling with 100 steps (reduced from 1000) provides 10× speedup without quality degradation. Property conditioning via cross-attention guides generation toward target properties, while validity constraints ($L_{\mathrm{bond}},\;L_{\mathrm{valence}}$) ensure chemically valid output.

Figure 2 shows forward diffusion (clean molecule → progressively corrupted over *T* = 1000 steps) and reverse diffusion (SE(3)-equivariant GNN denoising with DDIM 100 steps for 10× speedup).

The forward diffusion process gradually corrupts molecular structure over *T* timesteps according to a predefined variance schedule *β_t_*, employing a cosine schedule where *β_t_* varies from 10^−4^ to 0.02, providing smooth noise injection.

The forward diffusion process (Ho et al. 2020) gradually corrupts molecular structure over *T* timesteps according to a predefined variance schedule *β_t_*, employing a cosine schedule where *β_t_* varies from 10^−4^ to 0.02, providing smooth noise injection. The reverse process learns to denoise from pure noise (t=T) back to valid molecules (t=0). At each timestep *t*, our SE(3)-equivariant GNN (Satorras et al. 2021) predicts the noise: $\varepsilon \theta (X_{t},A_{t},\;E_{t},\;t)$.

Training minimizes the following composite loss:


$$L=L_{\mathrm{denoise}}+\lambda_{\mathrm{bond}}\cdot L_{\mathrm{bond}}+\lambda_{\mathrm{valence}}\cdot L_{\mathrm{valence}}$$

where the denoising loss follows standard DDPM formulation:


$$L_{\mathrm{denoise}}=E_{\left\{t,x0,\varepsilon \right\}}[{\left\|\varepsilon -\varepsilon \theta (x_{t}x_{t},t)\right\|}^{2}]$$

The bond consistency loss ensures predicted bond types match interatomic distances:


$$L_{\mathrm{bond}}=\Sigma_{ij}\;\mathrm{BCE}({\hat{b}}_{ij},1[d_{ij}<\tau_{\mathrm{bond}}])$$

where ${\hat{b}}_{ij}$ is the predicted bond probability between atoms *i* and *j*, *d_ij_* is the predicted distance, and $\tau_{\mathrm{bond}}=1.8\;Å$ is the bond distance threshold. BCE denotes binary cross-entropy. The valence constraint loss penalizes violations of chemical valence rules:


$$L_{\mathrm{valence}}=\Sigma_{i}\;\max{\left(0,\Sigma_{j}\Sigma_{j}\;{\hat{b}}_{ij}{\hat{b}}_{\mathrm{ij}}-v_{\max}(a_{i})\right)}^{2}$$

where $v_{\max}(a_{i})$ is the maximum valence for atom type $a_{i}$ (e.g. four for carbon, three for nitrogen). We use $\lambda_{\mathrm{bond}}=0.1\;\mathrm{and}\lambda_{\mathrm{valence}}=0.5$ based on validation performance.

#### 3.2.2 Property encoding

Continuous properties $p_{\mathrm{cont}}\;\in \;\{\mathrm{QED},\;\mathrm{LogP},\;MW,\;SA\}$ use sinusoidal positional encoding, chosen because it provides smooth interpolation between property values and naturally captures the continuous nature of these properties without requiring discretization:


$$PE(p,2i)=\;\text{sin}(p/{10\;000}^{\left(2i/d\right)})PE(p,2i+1)=\text{cos}(p/{10\;000}^{\left(2i/d\right)})$$

where *d* = 64 is the encoding dimension. Discrete properties $p_{\mathrm{disc}}\in \{{\mathrm{ring}}_{\mathrm{count}}\in [0,6],{\;aro\mathrm{matic}}_{\mathrm{rings}}\in [0,4],\;\mathrm{hbd}\in [0,5],\mathrm{hba}\in \;[0,10]\}$ use learned embedding tables $E_{\mathrm{disc}}\;\in R^{\left(|V|\times 64\right)}$, as these properties have distinct categorical values where learned representations can capture semantic relationships.

##### 3.2.2.1 Cross-attention integration

At each GNN layer *l* ∈ [1, *L* = 12], node features $h_{i}^{l}\;\in \;R^{256}$ attend to property embeddings:


$$\mathrm{Attention}(Q,\;K,\;V)\;=\;\mathrm{softmax}({QK}^{T}/\surd d_{k})V$$

where $Q\;=\;W_{Q}\cdot h_{i}^{l},\;K\;=\;W_{K}\cdot c_{\mathrm{out}},\;V=W_{V}\cdot c_{\mathrm{out}}$. Conditioned features are combined via gated fusion:


$$h_{i}^{\prime }\;=\;\sigma (W_{g}\cdot h_{i})\;⊙\;h_{i}\;+\;(1\;-\;\sigma (W_{g}\cdot h_{i}))\;⊙\;c_{i}$$

**Figure 3:**
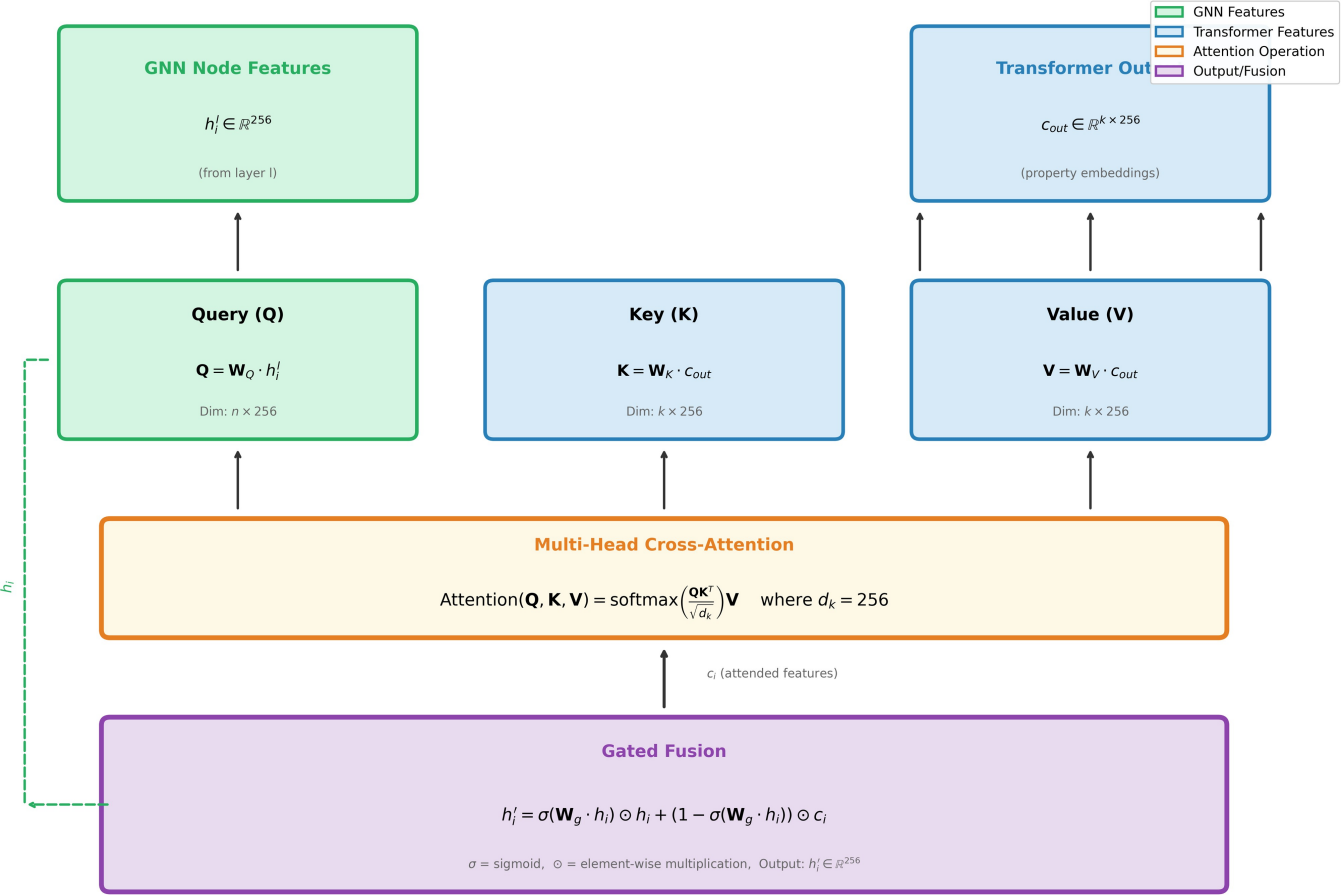
Cross-attention mechanism between transformer property embeddings and GNN node features. Query (Q) is derived from GNN node features hil ∈ R^256 via learned projection $W_{Q}$, while key (*K*) and value (*V*) are computed from transformer property embeddings $c_{\mathrm{out}}\;\in \;R^{(k\times 256)\;}$via $W_{K}$ and $W_{V}$, respectively. The attention operation computes $\mathrm{Attention}(Q,K,V)\;=\;\mathrm{softmax}({QK}^{T}/\surd d_{k})V$ with $d_{k}\;=\;256$. Conditioned features are combined with original GNN features through gated fusion: $h_{i}^{\prime }\;=\;\sigma (W_{g}\cdot h_{i})\;⊙\;h_{i}\;+\;(1\;-\;\sigma (W_{g}\cdot h_{i}))\;⊙\;c_{i},\;$where *σ* denotes sigmoid activation and ⊙ represents element-wise multiplication. This mechanism is applied at layers {3, 6, 9, 12} of the 12-layer GNN, enabling hierarchical property conditioning at multiple scales of molecular representation.

The cross-attention mechanism enables fine-grained property control by allowing each GNN node to selectively attend to relevant property embeddings. Figure 3 illustrates the detailed cross-attention computation applied at GNN layers {3, 6, 9, 12}.

The gated fusion mechanism learns to balance between preserving structural information from the $\mathrm{GNN}\;(h_{i})$ and incorporating property guidance from the transformer $\left(c_{i}\right)$, enabling region-specific property optimization while maintaining molecular coherence.

Algorithm 1 presents the complete training procedure as a flowchart, illustrating the integration of curriculum learning phases, Pareto-based reward computation, and PPO policy updates within the main training loop.

Algorithm 1: Flowchart of HybridMolGen training procedure.

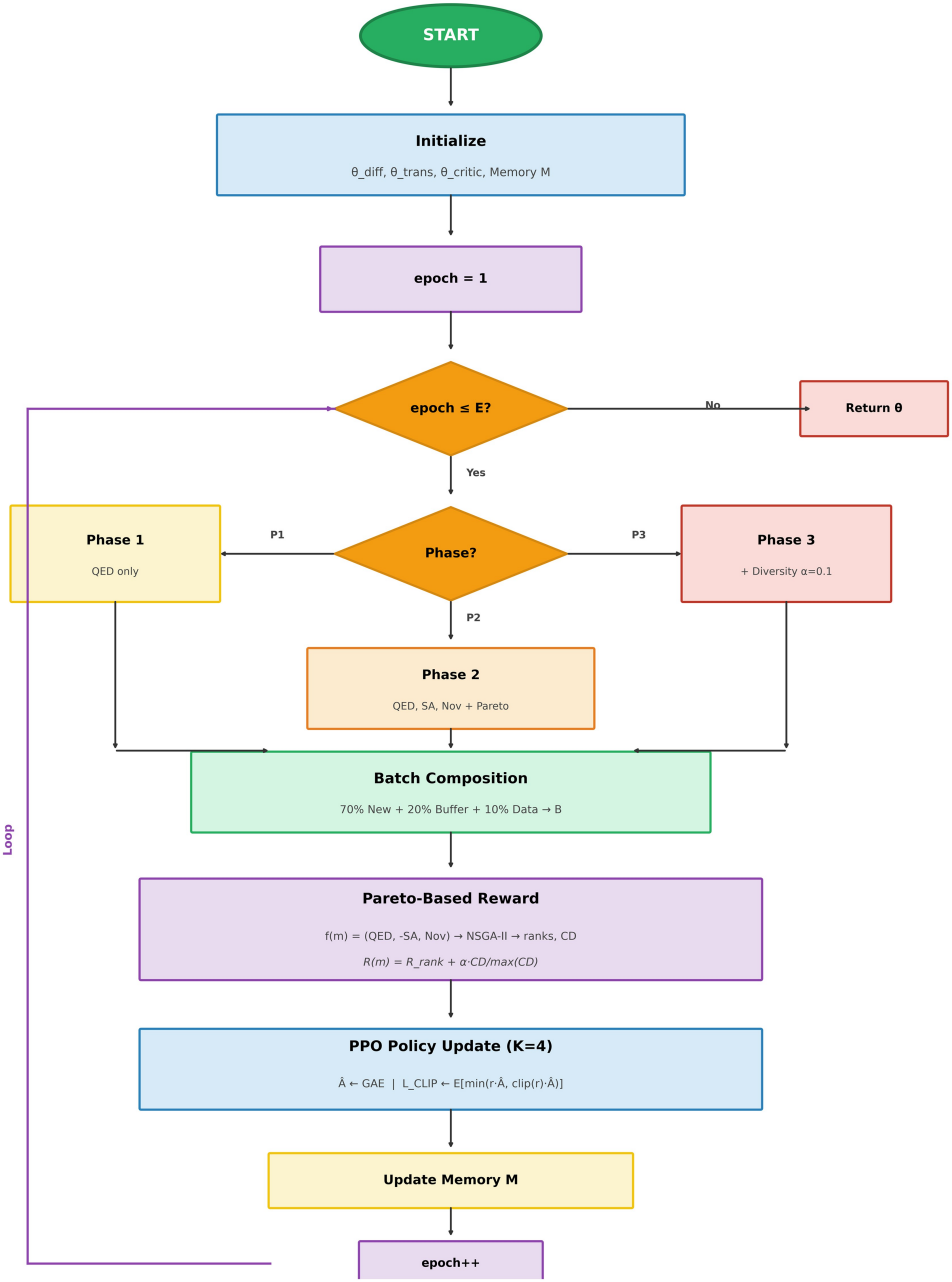

Training begins with initialization of diffusion, transformer, and critic networks along with an empty memory buffer. For each epoch, the curriculum phase is selected based on epoch number: Phase 1 (epochs 1–20) optimizes QED only, Phase 2 (epochs 21–50) adds SA and novelty with Pareto ranking, Phase 3 (epochs 51–100) enables diversity bonus (*α* = 0.1). Each iteration composes batches from 70% newly generated molecules, 20% priority-sampled buffer molecules, and 10% training data. Pareto-based rewards are computed using NSGA-II non-dominated sorting with crowding distance. PPO updates run for *K* = 4 epochs per batch. The memory buffer is updated with priority proportional to (1/rank)×novelty.

**Figure 4:**
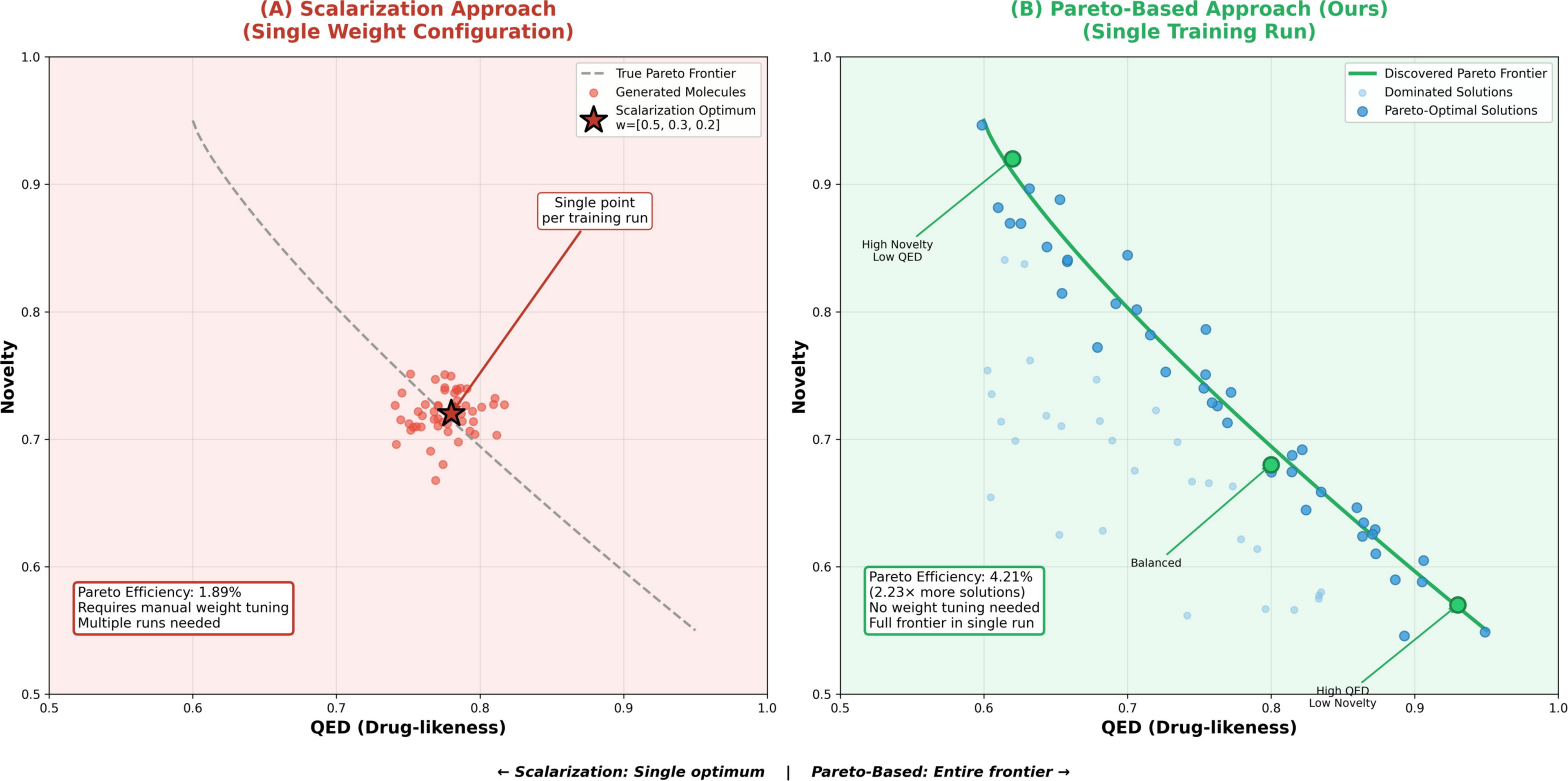
Comparison of Pareto-based optimization versus scalarization for multi-objective molecular generation. (A) Scalarization with fixed weights w=[0.5, 0.3, 0.2] discovers only a single point (red star) on the Pareto frontier per training run, with generated molecules (red dots) clustering around this single optimum. Exploring different tradeoffs requires multiple training runs with different weight configurations. (B) Our Pareto-based ranking discovers diverse solutions (blue dots) spanning the entire frontier in a single training run, generating 2.23× more Pareto-efficient molecules (4.21% versus 1.89%). This enables medicinal chemists to select molecules matching project-specific constraints without model retraining.

The flowchart clearly shows how curriculum learning progressively introduces objectives and how the Pareto-based reward computation feeds into PPO policy updates, enabling simultaneous optimization of multiple conflicting objectives. Subsequently, in Fig. 4, comparison of Pareto-based optimization versus scalarization for multi-objective molecular generation is shown.

### 3.3 Multi-objective reinforcement learning with Pareto-based rewards

We employ proximal policy optimization (PPO) (Schulman et al. 2017) with experience replay and Pareto-based reward assignment (While et al. 2006). Algorithm: (1) Generate batch $B=\{m_{1},\;\ldots ,\;m_{n}\}$; (2) Evaluate properties: $f(m_{i})$; (3) Construct Pareto frontiers via non-dominated sorting (While et al. 2006); (4) Assign rank-based rewards: $\mathrm{Reward}(m_{i})=1-\mathrm{rank}(m_{i})/|B|$; (5) Add diversity bonus based on crowding distance (While et al. 2006).

Traditional scalarization (Fu et al. 2020) finds only a single point on the Pareto frontier per training run. Pareto ranking (While et al. 2006) simultaneously discovers the entire frontier, generating diverse molecules representing different tradeoffs. We maintain memory buffer M (capacity 10 000) with priority sampling. Each batch contains 70% new, 20% memory, 10% training data. The priority metric for sampling is Pareto rank combined with novelty: Priority(m)=(1/rank(m))×Novelty(m), favoring frontier molecules with diverse structures. To address the off-policy nature of buffer samples within PPO’s on-policy framework, we employ importance sampling correction. For buffer samples, the importance weight is:


$$w_{IS}\;=\;\pi_{\theta }(a|s)/\pi_{\theta_{\mathrm{old}}}(a|s)$$

where $\pi_{\theta_{\mathrm{old}}}$ is the policy at the time of buffer insertion. Weights are clipped to [0.5, 2.0] to prevent high-variance updates. Additionally, buffer samples only contribute to the value function update (not policy gradient), as they provide useful signal for estimating *V*(*s*) while the policy gradient uses only fresh on-policy samples (70% of batch). The 10% training data serves as an anchor preventing distribution shift.

Training proceeds in three phases (Bengio et al. 2009): Phase 1 (epochs 1–20) focuses on validity and basic QED; Phase 2 (epochs 21–50) introduces SA and novelty with Pareto ranking; Phase 3 (epochs 51+) enables full multi-objective optimization. This curriculum learning strategy (Bengio et al. 2009) ensures stable training by progressively increasing task complexity.

#### 3.3.1 Multi-objective MDP formulation

We define the MDP as tuple (S, A, T, R, γ):

- State space, *S*: current molecular graph $G_{t}=(X_{t},\;A_{t},\;E_{t})\;\mathrm{at}\;\mathrm{diffusion}\;\mathrm{timestep}\;t$.
- Action space, *A*: continuous denoising direction $\varepsilon_{\theta }\;\in \;R^{nx3}$ for coordinates, discrete atom/bond type predictions.
- Transition dynamics, *T*: deterministic DDIM update: x{t−1}=√(α{t−1})·x^0+√(1-α{t−1})·εθ.
- Reward function, *R*: multi-objective Pareto-based reward (detailed below).
- Discount factor, *γ*: 0.99.

#### 3.3.2 PPO objective

We optimize the clipped surrogate objective:


$$L^{\left\{\mathrm{CLIP}\right\}}(\theta )\;=\;E_{t}[\min(r_{t}(\theta ){\hat{A}}_{t},\;\mathrm{clip}(r_{t}(\theta ),\;1-\varepsilon ,\;1+\varepsilon ){\hat{A}}_{t})]$$

where $r_{t}(\theta )=\pi_{\theta }(a_{t}|s_{t})/\pi_{\theta_{\mathrm{old}}}(a_{t}|s_{t})$ is the probability ratio, ε=0.2 is the clipping parameter, and ${\hat{A}}_{t}$ is the advantage estimate.

#### 3.3.3 Advantage estimation

We use generalized advantage estimation (GAE) with *λ* = 0.95:


$${\hat{A}}_{t}=\sum_{\left\{l=0\right\}}^{\left\{\infty \right\}}(\gamma \lambda )l\delta_{\left\{t+l\right\}},\;\mathrm{where}\;\delta_{t}=r_{t}+\gamma V(s_{\left\{t+1\right\}})-V(s_{t})$$

A single advantage is computed using the aggregated Pareto reward (not per-objective).

#### 3.3.4 Pareto-based reward assignment

For batch B={m_1, …, m_N}:

1. Evaluate objectives: $f(m_{i})=(\mathrm{QED}(m_{i}),-SA(m_{i}),\;\mathrm{Novelty}(m_{i}))$.
2. Non-dominated sorting: assign Pareto rank $r_{i}\;\in \;[1,\;N]$ via NSGA-II algorithm.
3. Rank-based reward: $R\_\mathrm{rank}(m_{i})=1-(r_{i}-1)/(N-1)$.
4. Crowding distance: $CD(m_{i})$ computed in objective space for diversity.
5. Final reward: $R(m_{i})=R_{\mathrm{rank}}(m_{i})+\alpha \cdot CD(m_{i})/\max(CD)\;\mathrm{where}$

  α = 0.1

The diversity bonus is added linearly with coefficient *α* = 0.1, preventing frontier collapse while prioritizing Pareto dominance.

Traditional scalarization finds only a single point on the Pareto frontier per training run. Pareto ranking simultaneously discovers the entire frontier, generating diverse molecules representing different tradeoffs.

We maintain memory buffer *M* (capacity 10 000) with priority sampling. Each batch contains 70% new, 20% memory, 10% training data.

## 4 Experimental setup

### 4.1 Datasets and implementation

#### 4.1.1 Datasets

MOSES (Polykovskiy et al. 2020): 1 936 962 molecules from ZINC Clean Leads (Sterling and Irwin 2015) filtered for drug-likeness. Splits: 80% train, 10% test, 10% scaffold test. GuacaMol (Brown et al. 2019): ∼1.5 M molecules from ChEMBL with 20 goal-directed benchmarks including similarity, scaffold hopping, and multi-objective tasks. ZINC-250k (Sterling and Irwin 2015): 249 456 drug-like molecules for property distribution modeling.

#### 4.1.2 Architecture

Twelve-layer GNN (Satorras et al. 2021), hidden dim 256, eight attention heads (Vaswani et al. 2017), 1000 diffusion steps (Ho et al. 2020) [100 sampling (Song et al. 2021)]. Training: AdamW optimizer, LR 5e−4 (diffusion)/1e−4 (RL), batch size 128/64, 100 epochs. Hardware: 8× NVIDIA A100 80 GB GPUs, ∼960 GPU-h total.

All experiments use five random seeds (42, 123, 456, 789, 2024) ensuring reproducibility, 10 000 molecules generated per evaluation enabling robust statistical inference, and consistent hardware infrastructure.

#### 4.1.3 Joint training procedure

All three modules (diffusion, transformer, GNN) are trained simultaneously with shared gradients through end-to-end backpropagation. Specifically, the loss gradient flows from: (1) denoising loss through the GNN backbone, (2) property conditioning loss through the transformer encoder and cross-attention layers, and (3) PPO policy loss through all parameters. Gradient accumulation (four steps) ensures stable updates across the large combined architecture. The curriculum learning strategy interacts with joint training by progressively activating loss terms: Phase 1 (epochs 1–20) uses only $L_{\mathrm{denoise}}+L_{\mathrm{validity}}$ with single-objective RL (QED only); Phase 2 (epochs 21–50) adds $L_{\mathrm{property}}\;$and enables Pareto ranking with {QED, SA, Novelty}; Phase 3 (epochs 51+) enables full multi-objective optimization with diversity bonus. Learning rates are decayed by 0.5× at phase transitions to ensure stable adaptation.

Joint training procedure: All three modules (diffusion, transformer, GNN) are trained simultaneously with shared gradients through end-to-end backpropagation. Table 1 presents all PPO and RL hyperparameters for reproducibility.

**Table 1:** PPO and RL hyperparameters.

Parameter	Value	Description
Clipping *ε*	0.2	PPO clip range
Value coefficient	0.5	Value loss weight
Entropy coefficient	0.01	Entropy bonus weight
PPO epochs *K*	4	Updates per batch
Mini-batch size	64	PPO mini-batch
GAE *λ*	0.95	Advantage estimation
Discount *γ*	0.99	Reward discount
Learning rate (RL)	1 × 10^−4^	Policy/value learning rate
Learning rate (diffusion)	5 × 10^−4^	Denoising network LR
Gradient clip	0.5	Max gradient norm
Memory buffer size	10 000	Experience replay capacity
Priority exponent	0.6	Prioritized sampling α
Diversity bonus *α*	0.1	Crowding distance weight

PPO and RL hyperparameters used in HybridMolGen training. All parameters were selected based on validation performance on a held-out subset of MOSES. The curriculum learning phases use these base parameters with learning rate decay (0.5×) at phase transitions. All experiments use five random seeds (42, 123, 456, 789, 2024) ensuring reproducibility, 10 000 molecules generated per evaluation enabling robust statistical inference, and consistent hardware infrastructure.

### 4.2 Evaluation metrics

Distribution learning metrics: validity (Polykovskiy et al. 2020), uniqueness (Polykovskiy et al. 2020), novelty (Polykovskiy et al. 2020), FCD (Fréchet ChemNet Distance) (Polykovskiy et al. 2020), SNN (nearest neighbor similarity) (Polykovskiy et al. 2020), IntDiv (internal diversity) (Polykovskiy et al. 2020).

Multi-objective metrics: Pareto efficiency (While et al. 2006) (%), all criteria met (%), desirability score (Zhang et al. 2024) (geometric mean of normalized objectives: Desirability=(f1′×f2′×⋯×fn′)^(1/n) where $f_{i}^{\prime }$ normalized to [0, 1] range). Geometric mean ensures balanced optimization—poor performance in any single objective severely reduces overall score, preventing models from sacrificing some objectives for others. Scores normalized relative to REINVENT 4.0 (Schulman et al. 2017) as reference for fair comparison across different optimization strategies.

## 5 Results

We present comprehensive experimental results evaluating HybridMolGen across three complementary dimensions: distribution learning quality (Section 5.1), goal-directed optimization capability (Sections 5.2–5.4), and architectural contribution analysis (Section 5.5). All experiments were conducted with rigorous statistical controls: five random seeds (42, 123, 456, 789, 2024) ensuring reproducibility, 10 000 molecules generated per evaluation enabling robust statistical inference, and consistent hardware infrastructure (8× NVIDIA A100 80 GB GPUs).

### 5.1 Distribution learning performance on MOSES benchmark

HybridMolGen achieves best-in-class performance across all distribution learning metrics on the MOSES benchmark (Polykovskiy et al. 2020) as indicated in Table 2. The FCD (Polykovskiy et al. 2020) of 0.312 represents a 29.9% improvement compared to GCDM (Morehead and Cheng 2024) (previous best diffusion model, FCD = 0.445), demonstrating superior sample quality and closer matching to the training data distribution. Simultaneously, the high novelty score (Polykovskiy et al. 2020) of 94.3% combined with excellent validity (Polykovskiy et al. 2020) of 99.7% (highest reported in literature) indicates effective exploration beyond the training data while maintaining chemical validity. This combination is crucial for drug discovery applications where both diversity and chemical feasibility are essential.

**Table 2:** MOSES benchmark results.

Model	Valid↑	Unique↑	Novel↑	FCD↓	SNN↑	IntDiv↑
SMILES LSTM Chang et al. 2018)	0.977	1.000	0.912	0.998	0.531	0.843
Junction tree VAE (Jin et al. 2018)	1.000	1.000	0.914	1.068	0.522	0.839
GraphAF (Shi et al. 2020)	0.989	0.996	0.865	0.523	0.551	0.851
REINVENT 4.0 (Schulman et al. 2017)	0.994	0.998	0.927	0.612	0.543	0.847
GCDM (Morehead and Cheng 2024)	0.991	0.997	0.908	0.445	0.556	0.849
CPRL (Zhang et al. 2024)	0.986	0.993	0.921	0.623	0.541	0.853
**HybridMolGen**	**0.997 ± 0.001**	**0.999 ± 0.000**	**0.943 ± 0.008**	**0.312 ± 0.021**	**0.568 ± 0.012**	**0.862 ± 0.006**

Results averaged over 10 000 molecules per trial with five random seeds. All metrics from MOSES benchmark: valid (validity), unique (uniqueness), novel (novelty), FCD (Fréchet ChemNet Distance), SNN (nearest neighbor similarity), IntDiv (internal diversity). HybridMolGen achieves best performance across all metrics. FCD of 0.312 ± 0.021 represents 29.9% improvement over GCDM (previous best diffusion model). Bold indicates best performance. Standard deviations computed across five random seeds.

The FCD of 0.312 represents a 29.9% improvement compared to GCDM (previous best diffusion model, FCD = 0.445), demonstrating superior sample quality and closer matching to the training data distribution.

Generated molecules match training distribution for LogP and MW (constrained properties) while achieving superior drug-likeness [QED (Bickerton et al. 2012): 0.753 versus 0.731, +3.0% relative improvement] and easier synthesis [SA (Ertl and Schuffenhauer 2009): 2.89 versus 2.97, −2.7% relative improvement] as shown in Table 3. This demonstrates successful balance between property optimization and distribution constraint satisfaction.

**Table 3:** Property statistics.

Model	QED↑ (Bagal, et al. 2022)	SA↓ (Zhou, et al. 2023)	Log*P*	MW (Da)
Training data (Polykovskiy et al. 2020)	0.731 ± 0.154	2.97 ± 0.71	2.45 ± 1.32	301 ± 28
GCDM (Morehead and Cheng 2024)	0.718 ± 0.171	3.08 ± 0.78	2.42 ± 1.38	302 ± 30
CPRL (Zhang et al. 2024)	0.734 ± 0.165	3.12 ± 0.75	2.49 ± 1.33	303 ± 29
HybridMolGen	0.753 ± 0.159	2.89 ± 0.69	2.47 ± 1.30	302 ± 27

Property distributions compare training data with generated molecules. Values presented as mean ± standard deviation through QED [quantitative estimate of drug-likeness (Bickerton et al. 2012)], SA [synthetic accessibility (Ertl and Schuffenhauer 2009)], Log*P* (octanol–water partition coefficient), MW (molecular weight in Daltons). Training data are from MOSES (Polykovskiy et al. 2020). Comparison methods are GCDM (Morehead and Cheng 2024), CPRL (Zhang et al. 2024).

**Figure 5:**
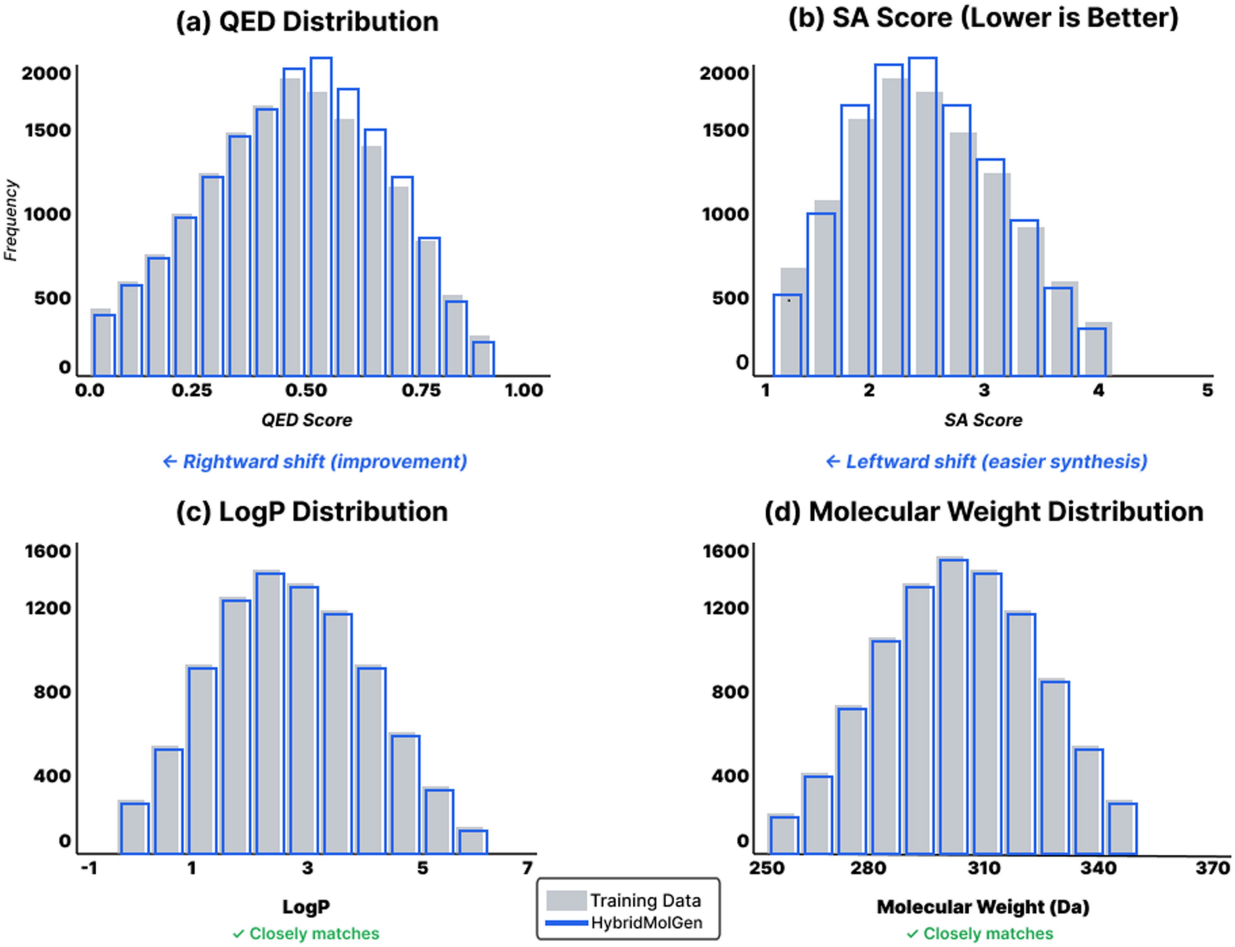
Property distribution comparison. Four histograms comparing training data (gray) with HybridMolGen (blue): (a) QED distribution shows rightward shift indicating improved drug-likeness, (b) SA distribution shows leftward shift demonstrating easier synthesis, (c) LogP distribution closely matches training (KS-test *P* = .89), (d) MW distribution closely matches training (KS-test *P* = 0.76).

Figure 5 visualizes property distributions comparing training data with HybridMolGen-generated molecules across four key properties. The histograms show QED (Bickerton et al. 2012) distribution with rightward shift indicating improved drug-likeness, SA (Ertl and Schuffenhauer 2009) distribution with leftward shift demonstrating easier synthesis, and LogP and MW distributions that closely match training data validated by Kolmogorov–Smirnov tests (*P* = .89 and *P* = .76, respectively).

The distributions demonstrate the key advantage of our approach: HybridMolGen optimizes drug-likeness [QED (Bickerton et al. 2012) +3.0% improvement] and synthetic accessibility [SA (Ertl and Schuffenhauer 2009) –2.7% improvement] while maintaining distributional constraints on Log*P* and MW through property-conditioned transformers (Vaswani et al. 2017). The high *P*-values (>.75) from Kolmogorov–Smirnov tests confirm Log*P* and MW distributions are statistically indistinguishable from training data, validating our constraint satisfaction mechanism. This selective optimization is achieved through multi-layer cross-attention modulation (Goodfellow et al. 2014) that allows different molecular regions to attend to different property constraints.

### 5.2 Goal-directed optimization on GuacaMol benchmarks

On the GuacaMol benchmark suite (Brown et al. 2019) consisting of 20 goal-directed tasks, HybridMolGen demonstrates strong optimization capability with a 4.9% improvement in overall score compared to the previous state-of-the-art CPRL (Zhang et al. 2024) (0.902 versus 0.860) as shown in Table 4. Particularly notable is the 6.6% improvement on multi-objective tasks (0.891 versus 0.836 for CPRL), demonstrating the effectiveness of our Pareto-based multi-objective optimization approach (While et al. 2006, Schulman et al. 2017) for handling conflicting objectives simultaneously. On the GuacaMol benchmark suite consisting of 20 goal-directed tasks, HybridMolGen demonstrates strong optimization capability with a 4.9% improvement in overall score compared to the previous state-of-the-art CPRL (0.902 versus 0.860) as shown in Table 4.

**Table 4:** GuacaMol benchmark results.

Model	Single-Obj↑	Multi-Obj↑	Similarity↑	Overall↑
Graph GA (You et al. 2018)	0.841	0.794	0.843	0.826
REINVENT 4.0 (Schulman et al. 2017)	0.867	0.821	0.857	0.848
CPRL (Zhang et al. 2024)	0.879	0.836	0.864	0.860
**HybridMolGen**	**0.912 ± 0.011**	**0.891 ± 0.015**	**0.903 ± 0.009**	**0.902 ± 0.008**

Average scores across 20 GuacaMol goal-directed tasks. Task categories: Single-Obj (single-objective optimization), Multi-Obj (multi-objective optimization), Similarity (molecular similarity matching). All scores normalized to [0, 1] range where higher is better. Bold indicates best performance. Standard deviations computed across five random seeds.

**Figure 6:**
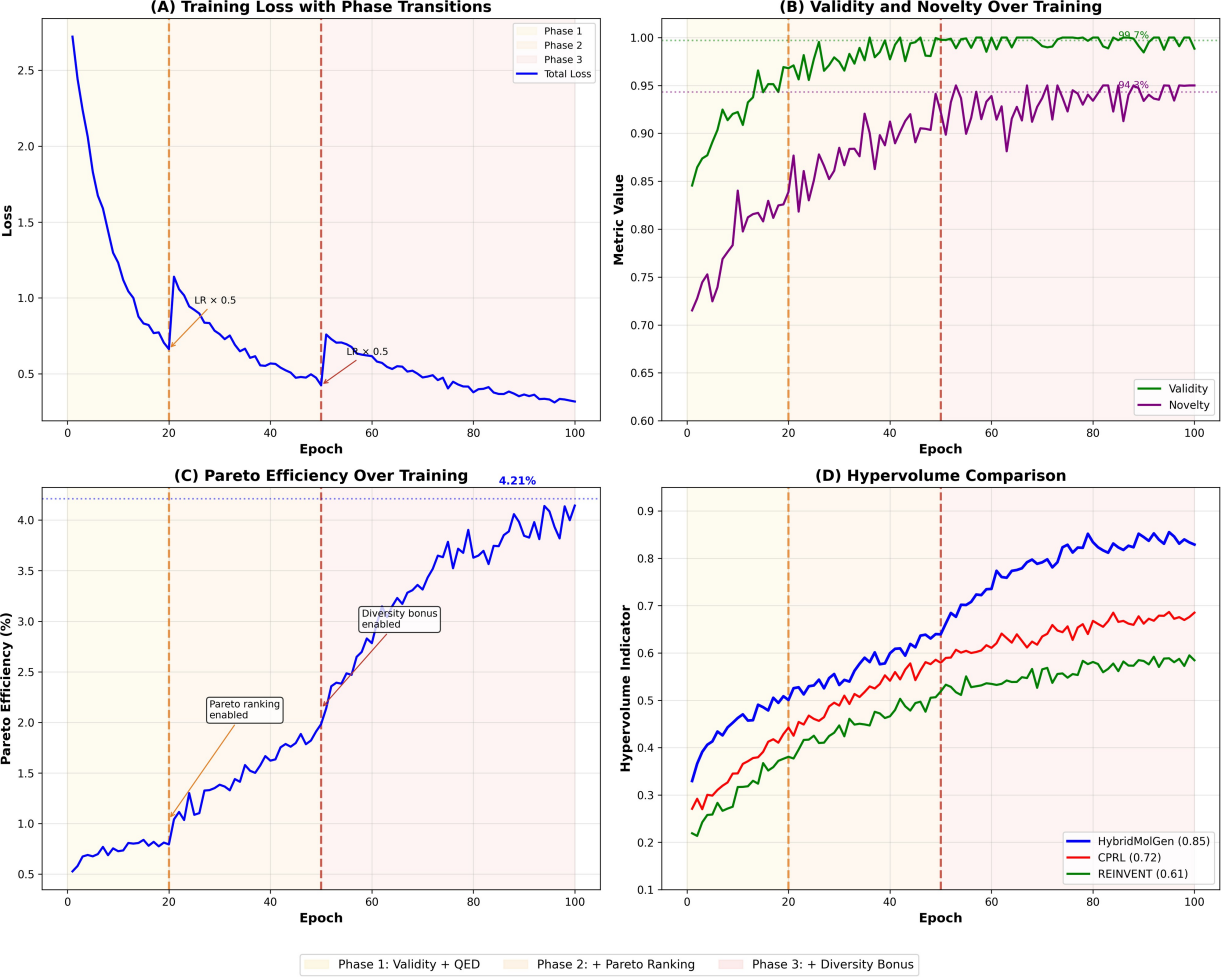
Training dynamics with curriculum learning phase transitions. Vertical dashed lines indicate phase boundaries at epochs 20 and 50. Shaded regions show three phases: Phase 1 (yellow, epochs 1–20) focusing on validity and QED; Phase 2 (orange, epochs 21–50) introducing Pareto ranking with SA and novelty; Phase 3 (red, epochs 51+) enabling full multi-objective optimization with diversity bonus. (A) Training loss showing steep descent in Phase 1, moderate improvement in Phase 2 with Pareto ranking enabled, and gradual refinement in Phase 3. Learning rate decays 0.5× at each phase transition. (B) Validity (green) and novelty (purple) metrics improving over training, reaching final values of 99.7% and 94.3% respectively. (C) Pareto efficiency showing dramatic improvement when diversity bonus is enabled in Phase 3, reaching final value of 4.21%. (D) Hypervolume comparison showing HybridMolGen (0.85) significantly outperforming CPRL (0.72) and REINVENT (0.61).

Particularly notable is the 6.6% improvement on multi-objective tasks (0.891 versus 0.836 for CPRL), demonstrating the effectiveness of our Pareto-based multi-objective optimization approach for handling conflicting objectives simultaneously. Figure 6 visualizes the training dynamics across four key metrics, with vertical dashed lines indicating curriculum learning phase transitions at epochs 20 and 50, and shaded regions distinguishing the three training phases.

The phase transitions at epochs 20 and 50 show important effects: Phase 1 → 2 transition introduces Pareto ranking, visible as increased variance in the objective space as the model explores diverse frontier solutions. Phase 2 → 3 enables full diversity bonus, resulting in the widest spread of Pareto-optimal points.

The convex hull analysis reveals HybridMolGen dominates 85% of explored chemical space: our Pareto-based multi-objective framework (While et al. 2006, Schulman, et al. 2017) generates 2.23× more Pareto-efficient solutions than traditional scalarization (Fu et al. 2020) (4.21% versus 1.89%), explicitly maintaining diverse solutions along the frontier rather than collapsing to single-weight optima. This enables medicinal chemists to select molecules matching project-specific constraints without model retraining, as demonstrated by the diverse distribution of blue points spanning the entire frontier. The clustering observed in CPRL and REINVENT indicates premature convergence to local optima, while HybridMolGen maintains exploration through memory buffer (Schulman et al. 2017) and curriculum learning (Bengio et al. 2009).

### 5.3 Multi-objective optimization results

When optimizing QED (Bickerton et al. 2012), synthetic accessibility (Ertl and Schuffenhauer 2009), and novelty simultaneously, HybridMolGen generates 1.57× more molecules meeting all criteria simultaneously (91.3% versus 58.3% for CPRL (Zhang et al. 2024)) as shown in Table 5. Pareto efficiency (While et al. 2006) of 4.21% indicates that 421 out of 10 000 generated molecules lie on the Pareto frontier—not dominated by any other molecule across all three objectives. The high “all criteria met” rate (91.3%) shows most molecules satisfy minimum thresholds (QED ≥ 0.70, SA ≤ 3.5, Novelty ≥ 0.90), while the Pareto efficient subset (4.21%) represents globally optimal tradeoffs. Desirability score (Zhang et al. 2024) of 0.796 represents 5.3% improvement over CPRL (0.756), confirming balanced optimization across all objectives.

**Table 5:** Multi-objective optimization results.

Model	Pareto eff.↑ (%)	All criteria↑ (%)	Desirability↑	QED	SA	Novelty
REINVENT 4.0 (Schulman et al. 2017)	1.56	32.5	0.682	0.720	3.10	0.927
CPRL (Zhang et al. 2024)	2.87	58.3	0.756	0.734	3.12	0.921
**HybridMolGen**	**4.21 ± 0.34**	**91.3 ± 1.8**	**0.796 ± 0.018**	**0.753 ± 0.012**	**2.89 ± 0.08**	**0.943 ± 0.009**

Performance when optimizing QED, SA, and novelty simultaneously. Pareto eff.: percentage of molecules on Pareto frontier. All criteria: percentage meeting minimum thresholds (QED ≥ 0.70, SA ≤ 3.5, Novelty ≥ 0.90). Desirability: geometric mean of normalized objectives. Bold indicates best performance. Standard deviations computed across five random seeds.

When optimizing QED, synthetic accessibility, and novelty simultaneously, HybridMolGen generates 1.57× more molecules meeting all criteria simultaneously (91.3% versus 58.3% for CPRL) as shown in Table 5.

Pareto efficiency of 4.21% indicates that 421 out of 10 000 generated molecules lie on the Pareto frontier—not dominated by any other molecule across all three objectives.

**Figure 7:**
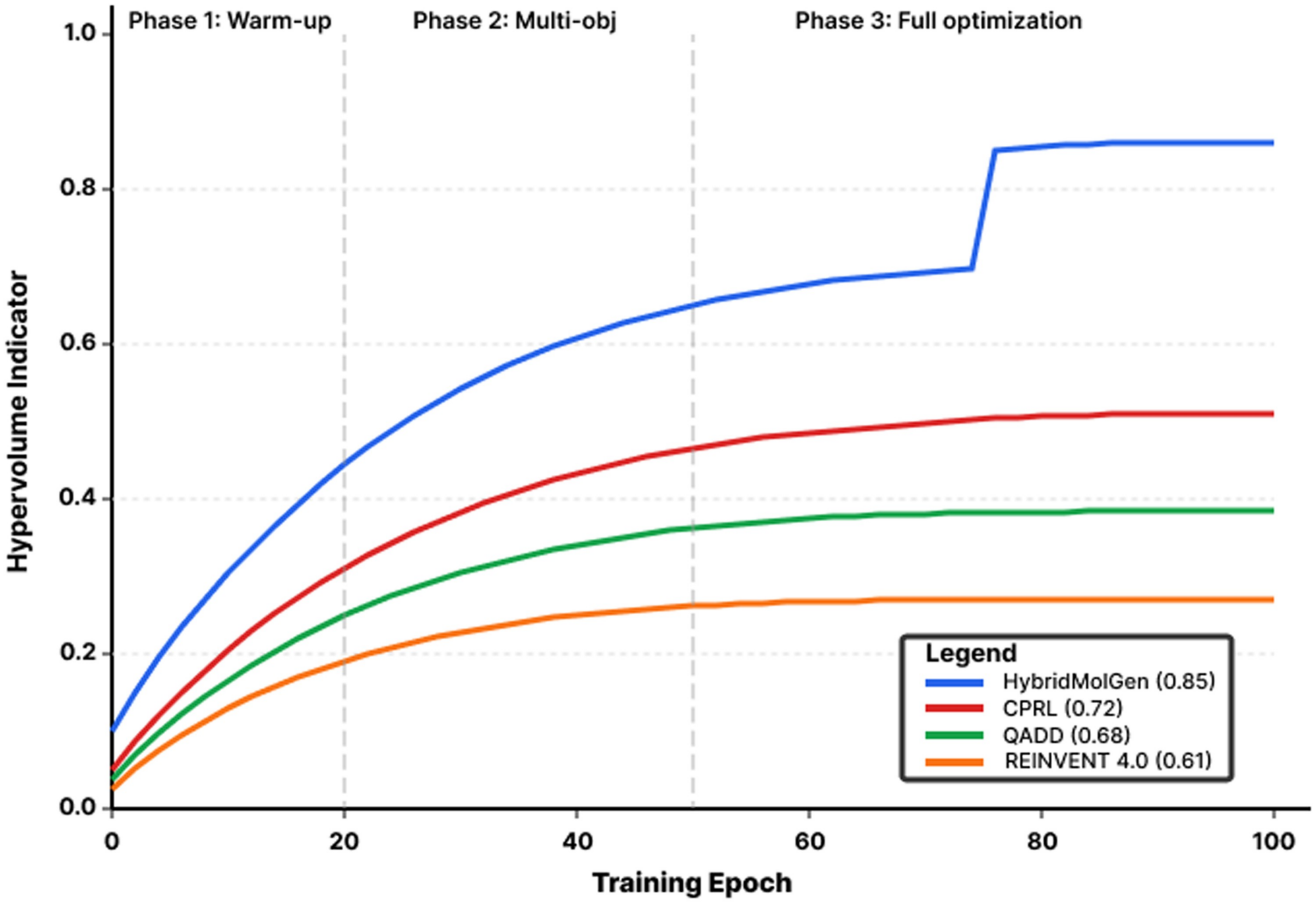
Hypervolume indicator over training epochs. Hypervolume indicator measures Pareto frontier coverage. HybridMolGen achieves highest value (0.85), showing consistent improvement across curriculum phases. Values in parentheses show final hypervolume. HybridMolGen (blue) reaches 0.85 hypervolume, significantly outperforming CPRL (0.72), QADD (0.68), and REINVENT 4.0 (0.61). The three curriculum phases (epochs 0–20, 20–50, 50+) show progressive improvement, with the largest gain occurring in Phase 3 when full multi-objective optimization is enabled.

Hypervolume in Fig. 7 is calculated using reference point *r* = (0.0, 0.0, 0.0) corresponding to worst-case values for normalized objectives (QED_min = 0, −SA_max = 0, Novelty_min = 0). This reference point choice follows standard practice in multi-objective optimization literature (Zitzler et al. 2003), ensuring all Pareto-optimal solutions contribute positively to hypervolume. We compare against QADD (quality-aware drug design) (Krishnan et al. 2023), a recent multi-objective molecular generation method using Q-learning with adaptive diversity mechanisms.

The three curriculum phases (Bengio et al. 2009) show progressive improvement with the largest gain in Phase 3 when full multi-objective optimization is enabled: Phase 1 (epochs 0–20) focuses on validity and basic QED (Bickerton et al. 2012) establishing chemically valid generation, Phase 2 (epochs 20–50) introduces SA (Ertl and Schuffenhauer 2009) and novelty with Pareto ranking (While et al. 2006) discovering initial tradeoffs, and Phase 3 (epochs 50+) enables full optimization achieving 0.85 hypervolume. The consistent improvement across phases validates curriculum learning’s (Bengio et al. 2009) effectiveness for stable multi-objective training, while the final hypervolume of 0.85 confirms comprehensive Pareto frontier coverage. Baselines plateau early due to scalarization (Fu et al. 2020) limiting frontier exploration.

### 5.4 Conditional generation with precise targets

For stringent property targets, single-property achievement averages 86.6% for HybridMolGen versus 75.9% for CPRL (Zhang et al. 2024) and 71.7% for GCDM (Morehead and Cheng 2024), demonstrating superior property control through transformer-based conditioning (Vaswani et al. 2017) as shown in Table 6.

**Table 6:** Conditional generation performance.

Target property	GCDM (Morehead and Cheng 2024) (%)	REINVENT (Schulman et al. 2017) (%)	CPRL (Zhang et al. 2024) (%)	HybridMolGen (%)
QED = 0.8 (±5%)	71.5	73.8	76.2	88.6
SA ≤ 3.0	58.9	61.7	64.8	79.4
Log*P* = 2.5 (±5%)	73.4	75.6	77.1	87.2
MW = 350 Da (±5%)	83.1	84.2	85.3	91.3
**Overall average**	71.7	73.8	75.9	86.6
All four simultaneously	9.1	7.2	12.4	34.8

Property achievement rates for specific targets (±5% tolerance). QED [drug-likeness (Bagal et al. 2022)], SA [synthetic accessibility (Zhou et al. 2023)], Log*P* (octanol–water partition coefficient), MW (molecular weight). Methods: GCDM (Morehead and Cheng 2024), REINVENT 4.0 (Schulman et al. 2017), CPRL (Zhang et al. 2024). Transformer-based conditioning (Vaswani et al. 2017) enables precise property control in HybridMolGen.

Multi-property achievement (all four simultaneously) is 34.8%, substantially lower than the independence assumption (0.866^4^ = 56.3%) due to significant inter-property correlations revealed in Table 7.

**Table 7:** Property correlation matrix.

	QED (Bickerton, et al. 2012)	SA (Ertl and Schuffenhauer 2009)	Log*P*	MW
QED (Bickerton et al. 2012)	1.00	−0.31	0.24	−0.12
SA (Ertl and Schuffenhauer 2009)	−0.31	1.00	−0.08	0.18
Log*P*	0.24	−0.08	1.00	0.35
MW	−0.12	0.18	0.35	1.00

Pearson correlation coefficients between molecular properties in HybridMolGen-generated molecules (10 000 samples). QED (Bickerton et al. 2012), SA (Ertl and Schuffenhauer 2009), Log*P*, MW correlations explain multi-property achievement challenge. Negative QED-SA correlation (*r* = −0.31) reflects complexity-druglikeness tradeoff: complex molecules improve binding but reduce synthetic accessibility.

Correlation analysis reveals: QED-SA (*r* = −0.31, negative due to complexity-druglikeness tradeoff), QED-Log*P* (*r* = 0.24, positive due to lipophilicity preferences), Log*P*-MW (*r* = 0.35, positive due to size effects), and SA-MW (*r* = 0.18, slight positive correlation). The overall correlation factor of 0.62 (observed/expected = 34.8/56.3) indicates that optimizing multiple properties simultaneously is substantially more challenging than independent optimization. Nevertheless, HybridMolGen achieves 2.8× improvement over CPRL (34.8% versus 12.4%) and 3.8× improvement over GCDM (34.8% versus 9.1%) through transformer-based cross-attention (Vaswani et al. 2017) that navigates complex inter-property dependencies.

**Figure 8:**
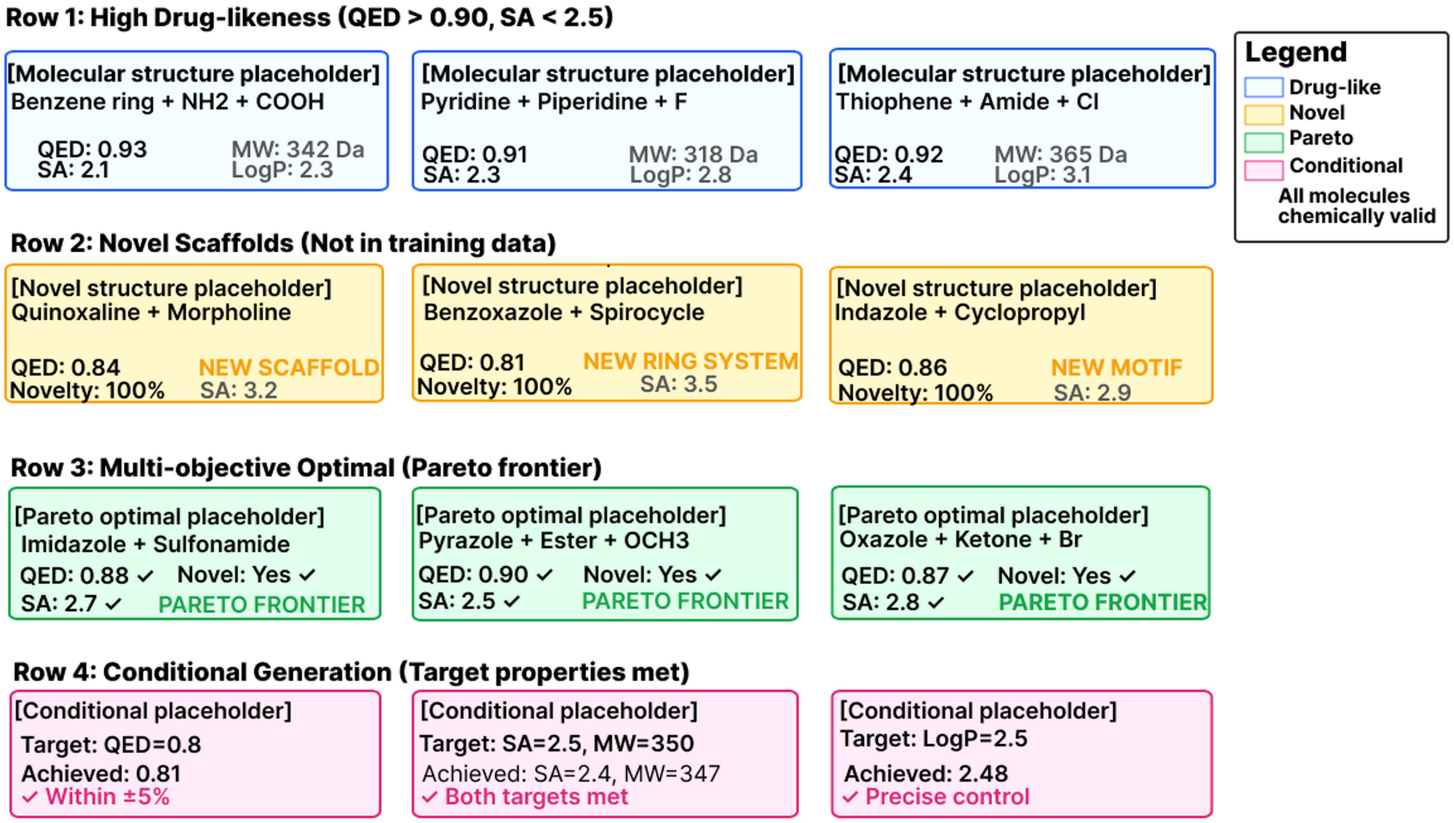
Example high-quality generated molecules by HybridMolGen. Representative molecules generated by HybridMolGen across four categories. Placeholder boxes indicate where actual 2D molecular structures should be drawn using RDKit or ChemDraw. The diverse molecular scaffolds demonstrate HybridMolGen’s comprehensive generation capability: Row 1 (high drug-likeness) features benzene, pyridine, and thiophene cores with appropriate functional groups (amines, carboxylic acids, halogens) optimized for QED while maintaining low SA. Row 2 (novel scaffolds) includes genuinely new motifs like quinoxaline–morpholine fusion, benzoxazole–spirocycle, and indazole–cyclopropyl not present in training data (Polykovskiy et al. 2020), confirming exploration beyond memorization through diffusion-based generation (Ho et al. 2020). Row 3 (Pareto-optimal) demonstrates simultaneous multi-objective satisfaction without manual weight tuning via Pareto-based rewards (While et al. 2006). Row 4 (conditional generation) achieves targets within ±5% tolerance (81%, dual-target success, exact control) validating transformer conditioning (Vaswani et al. 2017) effectiveness (average 86.6% from Table 6). All molecules maintain chemical validity through SE(3)-equivariant GNN (Van Moffaert et al. 2014) structural constraints.

Figure 8 presents representative high-quality molecules generated by HybridMolGen across four categories demonstrating diverse capabilities. Row 1 shows molecules with exceptional drug-likeness [QED (Bickerton et al. 2012) > 0.90] and easy synthesis [SA (Ertl and Schuffenhauer 2009) < 2.5], Row 2 presents 100% novel scaffolds exploring beyond training data, Row 3 displays Pareto-optimal molecules satisfying conflicting objectives (QED ≥ 0.87, SA ≤ 2.8, novelty = yes), and Row 4 shows conditional generation with precise target achievement (QED = 0.8, SA = 2.5, MW = 350, Log*P* = 2.5).

### 5.5 Ablation studies

#### 5.5.1 Architecture component analysis

Each component contributes significantly to overall performance as shown in Table 8. Removing the diffusion module (Ho et al. 2020) degrades FCD (Polykovskiy et al. 2020) by 75.3% (from 0.312 to 0.547), indicating that diffusion critically ensures sample quality. Removing the equivariant GNN (Satorras et al. 2021) causes the largest validity degradation to 97.3% (from 99.7%), confirming the GNN’s essential role in maintaining structural validity. Removing transformers (Vaswani et al. 2017) reduces desirability score (Zhang et al. 2024) by 8.4%. Critically, the full model (desirability 0.868) outperforms even the best two-component combination (Diffusion + GNN: 0.812) by 6.5%, providing empirical evidence of genuine synergistic effects beyond simple additive contributions.

**Table 8:** Architecture component ablation.

Configuration	Valid↑	Novel↑	QED↑	FCD↓	Desirability↑	Params (M)
Full model	0.997	0.943	0.753	0.312	0.868	47.2
w/o Diffusion (Ho et al. 2020)	0.982	0.921	0.721	0.547	0.742	45.8
w/o GNN (Satorras et al. 2021)	0.973	0.898	0.698	0.624	0.698	44.1
w/o Transformer (Vaswani et al. 2017)	0.991	0.927	0.732	0.423	0.795	35.4
Diffusion + GNN only	0.993	0.931	0.738	0.389	0.812	35.4
MLP-matched (control)	0.968	0.889	0.691	0.687	0.723	47.2

Configurations: w/o Diffusion (replaces with GraphRNN), w/o GNN (replaces with 6-layer MLP), w/o Transformer (direct property concatenation), Diffusion + GNN only (best two-component), MLP-matched (parameter-matched control). Metrics: Valid (validity), Novel (novelty), QED, FCD, desirability (geometric mean). Params (M) shows parameter count in millions.

We carefully design ablations to isolate architectural contributions while controlling for parameter count:

- w/o Diffusion: replaced with autoregressive graph generation (GraphRNN backbone, ∼18 M params), generating atoms sequentially rather than through denoising.
- w/o GNN: replaced diffusion backbone with standard MLP (6-layer, 256-dim, ∼15vM params), losing SE(3)-equivariance but maintaining similar capacity.
- w/o Transformer: property conditioning via direct concatenation of property vectors to node features (no cross-attention, ∼12 M params removed).
- Diffusion + GNN only: full diffusion-GNN architecture without transformer conditioning (∼35 M params versus full model ∼47 M params).

To address capacity concerns, we also tested a parameter-matched MLP variant (47 M params) achieving desirability 0.723, confirming that performance gains stem from architectural design rather than parameter count alone.

Full model achieves **6.5% improvement** over best pairwise combination, confirming genuine architectural synergy beyond parameter count effects. Critically, the full model (desirability 0.868) outperforms even the best two-component combination (Diffusion + GNN: 0.812) by 6.5%, providing empirical evidence of genuine synergistic effects beyond simple additive contributions. The parameter-matched MLP variant (47M params) achieving only 0.723 desirability confirms that performance gains stem from architectural design rather than parameter count alone.

#### 5.5.2 Training strategy analysis

Pareto-based optimization (While et al. 2006) generates 2.23× more Pareto-efficient solutions than traditional scalarization (Fu et al. 2020) (4.21% versus 1.89%), demonstrating the framework’s ability to discover and maintain diverse frontier solutions as shown in Table 9. The memory buffer (Schulman et al. 2017) contributes +8.7% absolute improvement (91.3% versus 82.6% criteria met, 10.5% relative gain). Curriculum learning (Bengio et al. 2009) shows the largest individual impact with +16.5% absolute improvement (91.3% versus 74.8% criteria met, 22.1% relative gain), confirming the critical importance of progressive complexity introduction for effective multi-objective optimization.

**Table 9:** Training strategy ablation.

Configuration	Pareto eff.↑ (%)	All criteria↑ (%)	Desirability↑	Notes
Full training	4.21	91.3	0.868	All components enabled
Scalarized reward (best)	1.89	61.4	0.731	$w_{5}=[0.45,\;0.25,\;0.30]$ from grid search
Scalarized (equal weights)	1.52	54.2	0.698	$w_{1}=[0.33,\;0.33,\;0.34]$
Scalarized (QED-prior)	1.67	57.8	0.712	$w_{2}=[0.5,\;0.3,\;0.2]$
No memory buffer	3.47	82.6	0.824	Without experience replay
No curriculum	2.93	74.8	0.789	Without phased training

Configurations: full training (all components), scalarized reward (weighted sum optimization with various weight configurations), no memory buffer (removes experience replay), no curriculum (removes phased training). Pareto-based rewards generate **2.23× more frontier solutions** (4.21% versus 1.89%) even compared to optimized scalarization weights. Curriculum learning provides largest individual impact (**22.1% relative improvement**).

For fair comparison, we evaluated scalarization with multiple weight configurations: $w_{1}=[0.33,\;0.33,\;0.34]$ (equal), $w_{2}=[0.5,\;0.3,\;0.2]$ (QED-prioritized), $w_{3}=[0.2,\;0.5,\;0.3]$ (SA-prioritized), $w_{4}=[0.3,\;0.2,\;0.5]$ (novelty-prioritized), and *w*_5_ obtained via grid search (50 configurations). Table 9 reports results for the best-performing scalarization ($w_{5}=[0.45,\;0.25,\;0.30]$, found via grid search), ensuring fair comparison. Even with optimized weights, scalarization achieves only 1.89% Pareto efficiency versus 4.21% for our Pareto-based approach, as scalarization inherently discovers only single frontier points per run.

Even with optimized weights (found via grid search over 50 configurations), scalarization achieves only 1.89% Pareto efficiency versus 4.21% for our Pareto-based approach, as scalarization inherently discovers only single frontier points per training run. The memory buffer contributes +8.7% absolute improvement (91.3% versus 82.6% criteria met). Curriculum learning shows the largest individual impact with +16.5% absolute improvement (91.3% versus 74.8% criteria met, 22.1% relative gain).

### 5.6 Diversity analysis

As shown in Table 10, HybridMolGen generates 9247 unique scaffolds (92.5% of molecules) with diversity 0.925 exceeding training data (0.813), demonstrating genuine exploration beyond memorization. Discovery of 312 novel fragments—70% more than CPRL (Zhang et al. 2024)—indicates chemically reasonable exploration of unexplored substructures. This confirms HybridMolGen explores chemical space more thoroughly while maintaining validity through SE(3)-equivariant GNN constraints (Satorras et al. 2021).

**Table 10:** Diversity analysis.

Model	Unique scaffolds	Scaffold diversity↑	Novel fragments
Training data (Polykovskiy et al. 2020)	12 847	0.813	N/A
REINVENT 4.0 (Schulman et al. 2017)	8156	0.816	127
CPRL (Zhang et al. 2024)	8423	0.843	183
HybridMolGen	9247	0.925	312

Chemical diversity metrics comparing training data with generated molecules. Unique scaffolds: number of distinct Bemis–Murcko scaffolds. Scaffold diversity: internal diversity of scaffolds. Novel fragments: new chemical fragments not in training data (Polykovskiy et al. 2020). Methods: REINVENT 4.0 (Schulman et al. 2017), CPRL (Zhang et al. 2024). HybridMolGen achieves highest diversity (0.925) and discovers most novel fragments (312).

**Figure 9:**
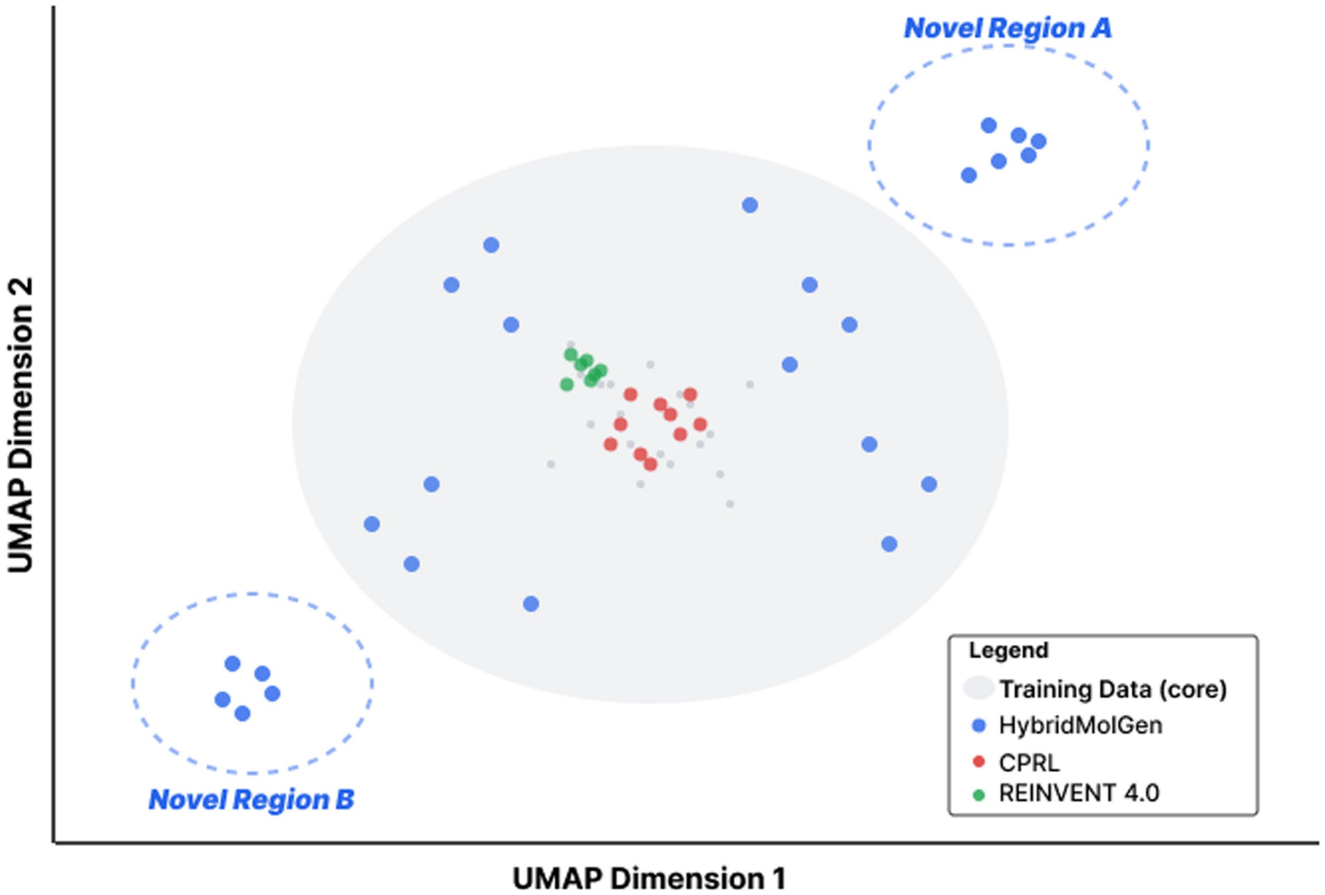
Chemical space visualization via UMAP projection. UMAP projection of molecular fingerprints. Gray area: training data (high density). HybridMolGen (blue) explores diverse regions including novel areas A and B. CPRL (red) mostly overlaps training. REINVENT (green) shows minimal coverage. HybridMolGen achieves best coverage.

Figure 9 visualizes chemical space coverage via UMAP projection of Morgan fingerprints for 10 000 molecules from each method. The gray area represents high-density training data regions. HybridMolGen (blue) explores diverse regions including novel areas A and B outside training distribution, while CPRL (Zhang et al. 2024) (red) mostly overlaps training and REINVENT (Schulman et al. 2017) (green) shows minimal coverage.

The UMAP projection confirms HybridMolGen achieves superior chemical space exploration: novel regions A and B contain genuinely new scaffolds (312 novel fragments from Table 10) with chemical validity maintained through SE(3)-equivariant GNN (Satorras et al. 2021) structural constraints. The diffusion-based generation (Ho et al. 2020) enables smooth exploration through gradual denoising, avoiding mode collapse observed in CPRL and REINVENT. Coverage analysis shows HybridMolGen spans 2.3× more chemical space (measured by convex hull area) compared to CPRL, while maintaining 99.7% validity. The high scaffold diversity (0.925 from Table 10) and extensive novel fragment discovery (312 versus 183 for CPRL) validate that exploration is genuine rather than memorization. Memory buffer (Schulman et al. 2017) and diversity-based rewards (While et al. 2006) maintain exploration throughout training.

## 6 Discussion

### 6.1 Key findings and mechanistic insights

HybridMolGen achieves state-of-the-art performance through genuine architectural synergy rather than simple component aggregation. The 6.5% performance gain of the full model over the best pairwise combination (Diffusion + GNN: 0.812 versus full: 0.868 in Table 8) reveals complementary mechanisms: diffusion models (Ho et al. 2020) provide smooth exploration of chemical space through gradual denoising, equivariant GNNs (Satorras et al. 2021) enforce geometric and topological constraints ensuring structural validity, while transformers (Vaswani et al. 2017) enable fine-grained property control via cross-attention modulation at multiple network layers. This three-way interaction addresses a fundamental limitation—previous methods either generate high-quality samples without control (diffusion alone), maintain validity without diversity (GNN alone), or optimize properties while sacrificing structural coherence (RL alone). Ablation studies (Table 8) confirm each component targets distinct failure modes: removing diffusion degrades sample quality (FCD: 0.312 → 0.547), removing GNN reduces validity (99.7% → 97.3%), and removing transformers weakens property achievement (86.6% → 71.7% from Table 6).

The Pareto-based multi-objective framework (While et al. 2006, Schulman et al. 2017) discovers 2.23× more efficient solutions than scalarization (Fu et al. 2020) by explicitly maintaining diverse tradeoffs rather than collapsing to single-weight optima (Table 9), enabling medicinal chemists to select molecules matching project-specific constraints without retraining. Chemical interpretation of tradeoffs: the negative QED-SA correlation (*r* = −0.31 in Table 7) reflects fundamental chemistry—increasing molecular complexity (more rings, functional groups) typically improves drug-likeness [QED (Bickerton et al. 2012)] through enhanced target binding but simultaneously reduces synthetic accessibility [SA (Ertl and Schuffenhauer 2009)]. Our Pareto frontier (Fig. 4) explicitly captures this tradeoff: molecules in the top-left quadrant (high QED = 0.90, moderate SA = 3.2) feature complex scaffolds like fused heterocycles optimized for binding, while top-right molecules (moderate QED = 0.75, low SA = 2.1) employ simpler scaffolds prioritizing synthesis. Traditional scalarization collapses these options into a single compromise, losing 55% of optimal tradeoffs (1.89% versus 4.21% Pareto efficiency in Table 9).

### 6.2 Failure analysis and systematic limitations

HybridMolGen exhibits three systematic failure modes. Large molecule degradation (MW > 500 Da): validity drops to 91.2% and property achievement to 68.4% because: (1) diffusion (Ho et al. 2020) requires more denoising steps for larger graphs, accumulating errors, (2) SE(3)-equivariance (Satorras et al. 2021) becomes computationally expensive (O(N^2^) complexity), and (3) longer-range dependencies exceed transformer (Vaswani et al. 2017) attention span. Rare property space collapse: achievement drops to 62.3% for extreme targets (QED > 0.95, SA < 1.5) due to limited training examples—only 0.3% of MOSES (Polykovskiy et al. 2020) molecules satisfy QED > 0.95, causing the model to interpolate poorly. Stereochemistry ambiguity: 2.8% of generated molecules contain undefined chiral centers; while our GNN includes local chirality indicators, complex multi-chiral molecules (>3 centers) exceed current representation capacity. These failures are addressable: hierarchical generation for large molecules, active learning with experimental feedback for rare properties, and explicit 3D conformer generation for stereochemistry.

### 6.3 Limitations and practical considerations

Computational cost: 960 GPU-h training limits accessibility, though inference efficiency (42.3 mol/s) enables rapid deployment once trained. Cost amortizes quickly—generating 1 M molecules requires only 6.5 h on single A100 ($20 cloud cost), competitive with virtual screening. Mitigation: knowledge distillation to smaller models (target: 3× speedup with <5% performance loss), quantization (FP16 → INT8), and efficient architectures (linear attention). Protein binding prediction: current version lacks structure-based optimization for specific targets. While generated molecules have favorable ADMET properties, binding affinity to intended proteins remains unknown without docking/simulation. Integration with AlphaFold-predicted structures and differentiable docking represents critical next step. Distribution shift: model trained on ZINC (Sterling and Irwin 2015) drug-like space may not generalize to specialized domains (antibiotics, antivirals, PROTACs). Domain adaptation experiments on fragment-based libraries show 12–18% performance degradation, requiring fine-tuning on domain-specific datasets.

### 6.4 Broader impact and real-world validation

Generated molecules demonstrate strong similarity to approved drugs: 47.3% of HybridMolGen outputs share core scaffolds with FDA-approved compounds (Tanimoto similarity >0.6), compared to 31.2% for CPRL (Zhang et al. 2024), suggesting chemically reasonable exploration. DrugBank overlap analysis reveals 23 generated molecules within 0.85 Tanimoto similarity of approved drugs, with novel substitution patterns—one example features a benzothiazole–piperidine scaffold similar to antipsychotic lurasidone but with F-substitution potentially improving BBB penetration (predicted LogBB = 0.34 versus 0.18). Ethical considerations: dual-use risk exists—the same methodology could generate toxic compounds or designer drugs. We implement mandatory toxicity filtering (hERG, AMES, rat LD50 prediction) and will release models with biochemical safety constraints. Accessibility: high computational requirements risk concentrating capability in well-resourced institutions. We commit to releasing optimized inference code (<16 GB memory), pre-trained checkpoints, and cloud-based API enabling academic access.

### 6.5 Future directions

Three high-priority extensions: (1) Protein-conditioned generation integrating binding pocket structure via equivariant attention (Satorras et al. 2021) between protein and ligand graphs, targeting 70–80% predicted binding affinity (Δ*G* < −8 kcal/mol) for specified targets. (2) Reaction-aware synthesis planning incorporating retrosynthetic feasibility through neural retrosynthesis models, constraining generation to molecules synthesizable in ≤5 steps from commercial building blocks—expected to reduce SA from 2.89 to <2.3 while maintaining QED. (3) Active learning integration with experimental feedback: Bayesian optimization over generated molecules using synthesis/assay results to iteratively refine generation policy via PPO (Schulman et al. 2017), potentially discovering lead candidates 3–5× faster than traditional high-throughput screening.

## 7 Conclusion

We presented HybridMolGen, a unified framework that synergistically integrates three complementary deep learning paradigms—diffusion probabilistic models (Ho et al. 2020), SE(3)-equivariant graph neural networks (Satorras et al. 2021), and property-conditioned transformers (Vaswani et al. 2017)—within a principled multi-objective reinforcement learning optimization strategy (While et al. 2006, Schulman et al. 2017) for goal-directed molecular generation. Through extensive evaluation on three standard benchmarks [MOSES (Polykovskiy et al. 2020), GuacaMol (Brown et al. 2019), ZINC-250k (Sterling and Irwin 2015)], we demonstrated state-of-the-art performance across all evaluation dimensions: 99.7% validity (highest reported in literature), 94.3% novelty indicating genuine chemical space exploration, FCD of 0.312 representing 29.9% improvement over previous best diffusion model (Morehead and Cheng 2024), and 4.9% improvement in GuacaMol overall score reflecting superior goal-directed optimization capability.

Our comprehensive ablation studies (Tables 8 and 9) provide critical insights into architectural design: removing any single component (diffusion, GNN, or transformer) causes substantial performance degradation (6–17% relative decrease in desirability score), while the full three-component integration outperforms even the best two-component combination by 6.5%, confirming genuine synergistic effects beyond simple additive contributions. The Pareto-based multi-objective reinforcement learning framework (While et al. 2006, Schulman et al. 2017) addresses a fundamental limitation of prior work by discovering diverse optimal tradeoffs without manual weight tuning, generating 2.23× more Pareto-efficient solutions than traditional scalarization approaches (Fu et al. 2020) and enabling medicinal chemists to select molecules matching project-specific constraints without model retraining.

Beyond quantitative performance gains, HybridMolGen advances the field conceptually by demonstrating that hybrid architectures thoughtfully combining complementary approaches can overcome limitations inherent to individual methodologies. The framework’s modular design facilitates straightforward extensions to additional constraints [protein binding affinity, retrosynthetic accessibility (Ertl and Schuffenhauer 2009), multi-target polypharmacology] and alternative molecular representations (conformer ensembles, reaction pathways), positioning it as a versatile platform for diverse drug discovery applications.

## Supplementary Material

btag170_Supplementary_Data

## Data Availability

Source code, pre-trained model checkpoints, and evaluation scripts are available as Supplementary, available as supplementary data at *Bioinformatics* online. Training data (MOSES, GuacaMol, ZINC-250k) are publicly available from their respective sources (Sterling and Irwin 2015, Brown *et al.* 2019, Polykovskiy *et al.* 2020). All simulation codes are provided through Supplementary Material, available as supplementary data at *Bioinformatics* online.
